# PRMT7 targets of Foxm1 controls alveolar myofibroblast proliferation and differentiation during alveologenesis

**DOI:** 10.1038/s41419-021-04129-1

**Published:** 2021-09-08

**Authors:** Huacheng He, Jilin Chen, Jian Zhao, Peizhun Zhang, Yulong Qiao, Huajing Wan, Jincheng Wang, Mei Mei, Shilai Bao, Qiuling Li

**Affiliations:** 1 State Key Laboratory of Molecular Developmental Biology, Institute of Genetics and Developmental Biology, the Innovative Academy of Seed Design, Chinese Academy of Sciences, Beijing, 100101 P.R. China; 2 Department of Health Sciences, Institute of Physical Science and Information Technology, Anhui University, Hefei, 230601 P.R. China; 3 Laboratory of Pulmonary Immunology and Inflammation, Department of Respiratory and Critical Care Medicine, Frontiers Science Center for Disease-related Molecular Network, West China Hospital, Sichuan University, Chengdu, Sichuan 610041 P.R. China; 4 School of Life Sciences, University of Chinese Academy of Sciences, Beijing, 100101 P.R. China

**Keywords:** Cell proliferation, Differentiation, Respiratory tract diseases

## Abstract

Although aberrant alveolar myofibroblasts (AMYFs) proliferation and differentiation are often associated with abnormal lung development and diseases, such as bronchopulmonary dysplasia (BPD), chronic obstructive pulmonary disease (COPD), and idiopathic pulmonary fibrosis (IPF), epigenetic mechanisms regulating proliferation and differentiation of AMYFs remain poorly understood. Protein arginine methyltransferase 7 (PRMT7) is the only reported type III enzyme responsible for monomethylation of arginine residue on both histone and nonhistone substrates. Here we provide evidence for PRMT7’s function in regulating AMYFs proliferation and differentiation during lung alveologenesis. In *PRMT7*-deficient mice, we found reduced AMYFs proliferation and differentiation, abnormal elastin deposition, and failure of alveolar septum formation. We further shown that oncogene forkhead box M1 (Foxm1) is a direct target of PRMT7 and that PRMT7-catalyzed monomethylation at histone H4 arginine 3 (H4R3me1) directly associate with chromatin of *Foxm1* to activate its transcription, and thereby regulate of cell cycle-related genes to inhibit AMYFs proliferation and differentiation. Overexpression of *Foxm1* in isolated myofibroblasts (MYFs) significantly rescued *PRMT7*-deficiency-induced cell proliferation and differentiation defects. Thus, our results reveal a novel epigenetic mechanism through which PRMT7-mediated histone arginine monomethylation activates *Foxm1* transcriptional expression to regulate AMYFs proliferation and differentiation during lung alveologenesis and may represent a potential target for intervention in pulmonary diseases.

## Introduction

Alveologenesis is the terminal step of lung development in which mature pulmonary gas exchange units (alveoli) are formed through a dramatic remodeling of the large, thick-walled primitive saccules [1, 2]. Abnormal alveologenesis has devastating effects and is often associated with chronic lung diseases such as bronchopulmonary dysplasia (BPD) [2–4]. The mechanisms involved in alveologenesis include the expansion of alveolar epithelium and alveolar myofibroblasts (AMYFs) localizing to the tip of crests to deposit elastin, which produce the necessary force for lifting the alveolar crest from the primary septa wall [5, 6]. AMYFs is a special type of fibroblast, which has the characteristics of fibroblast and smooth muscle cells in both ultrastructure and physiological function [7]. In the pseudoglandular phase of lung development, a group of PDGFRα-expressing interstitial cells around the distal epithelium began to appear, which were the precursors of AMYFs [8]. Later on, in the saccular phase, PDGFRα positive cells migrated to the periphery of the terminal cyst wall to form the secondary crest, and differentiated into mature α-SMA^+^ AMYFs to produce elastin [9]. Disruption of AMYFs differentiation, proliferation, or migration leads to the failure formation of the secondary septum and arrested alveolarization during lung development [10], and are found in several pulmonary diseases including emphysema, idiopathic pulmonary fibrosis (IPF), BPD, and chronic obstructive pulmonary disease (COPD) [11]. Moreover, given that AMYFs are the primary cell types responsible for the accumulation of extracellular matrix (ECM) during fibrotic diseases, targeting AMYFs proliferation and differentiation is an important therapeutic strategy for the treatment of pulmonary diseases [12].

AMYFs specification, proliferation, and migration are tightly regulated during lung development. Platelet-derived growth factor receptor α (PDGFRα) signaling is crucial for AMYFs differentiation and maturation [8, 9, 13]. It is reported that disruption of PDGFA in mice results in failure of elastic fiber deposition and secondary septum formation by repressing myofibroblast (MYF) differentiation [9]. Insulin-like growth factor 1 (IGF1) and transforming growth factor β1 (TGF-β1) are known to be required for AMYFs specification in mice. IGF1 promotes α-SMA expression and elastin deposition in MYFs of developing lung tissue, and overexpression of IGF1 enhances MYFs migration and proliferation in vitro [14]. TGF-β1 was reported to promote the differentiation of lung fibroblasts into MYFs, and deletion of *SMAD3*, an important intermediate signal molecule of TGF-β1, and results in failure of septa formation and decrease elastin deposition phenotype in mice [15]. In contrast to the well-established roles of these signaling factors, relatively little is known about how epigenetic mechanisms regulating the proliferation and differentiation of AMYFs during lung development and pulmonary diseases.

Protein arginine methyltransferase 7 (PRMT7) was reported as a type III enzyme that catalyze monomethylation and symmetrically di-methylation of arginine residues on both histone and nonhistone substrates to modulate diverse biological processes, including cell proliferation, carcinogenesis, and stem cell biology [16]. Our previous studies have shown that PRMT7 catalyzes monomethylation at histone H4 arginine 3 (H4R3me1) to negatively regulate the expression of Bcl6 during B-cell maturation [17]. To ensure timely activation of antiviral defense, PRMT7 is tightly controlled to suppress MAVS activation [18]. PRMT7 is also involved in the regulation of germ cell proliferation and myoblast differentiation, *PRMT7* deficiency causes impaired myogenic differentiation and defects in regenerative capacity upon muscle injury [19, 20]. PRMT7 is also reported to suppress cellular senescence through methylates GLI2 on arginine residues 225 and 227 in mouse embryonic fibroblasts (MEFs) [21]. Moreover, PRMT7 contributes to promoting metastasis in human breast cancer, non-small-cell lung cancer cells, and renal cell carcinoma [22–24], and it is increasingly being recognized as a potential drug target [24, 25].

In this study, we show that *PRMT7*-deficient mice exhibited enlarged distal airspaces secondary to the failure of alveolar septation, impaired AMYFs differentiation, proliferation, and elastin deposition. We also identified that PRMT7 and H4R3me1 directly associate with *Foxm1* to activate its transcription, and thereby enhance AMYFs proliferation and differentiation. Collectively, these findings represent one mechanistic explanation for how PRMT7 regulating AMYFs proliferation and differentiation during lung alveologenesis, and may provide potential clues for the treatment of pulmonary diseases caused by AMYF proliferation and differentiation disorders.

## Results

### Inactivation of PRMT7 disrupts alveolar development in mice

**Fig. 1:**
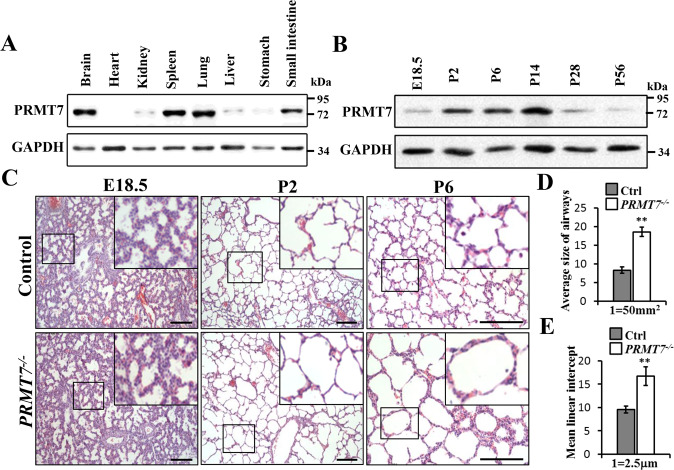
Impaired alveolarization in *PRMT7*^*−/−*^ lungs. **A** Western blot analysis showing the boundary expressed PRMT7 in lung tissues. **B** PRMT7 expression was significantly increased in lung tissues during P2 to P14. For (**A**) and (**B**), GAPDH serves as a loading control. The experiment was repeated three times with similar results. **C** Representative hematoxylin and eosin (H&E) staining showing severely enlarged alveolar airspace in *PRMT7*^−*/*−^ lungs at P2 and day P6. Boxed regions are magnified in insets. Scale bars: 100 μm. **D**, **E** Quantitated bar graphs showing the average size of distal airways (**D**) and the mean linear intercept (**E**), which describes the mean free distance in airspaces, was significantly larger in *PRMT7*^*−/−*^ lungs. Data were presented as mean ± SD. *n* = 8 mice per genotype. **P < 0.01 (Student’s *t*-test).

By analyzing the PRMT7 expression level in various tissues, we detected more abundant expressed PRMT7 in the lung, brain, stomach, and spleen tissues at postnatal day (P) 2 (Fig. 1A). We then examined the temporal expression level of PRMT7 at different lung developmental stages and found that the PRMT7 expression was notably increased from P2 to P14 (Fig. 1B), a time period when the parenchymal walls were undergoing the process of alveologenesis. Histological analysis revealed that whereas at embryonic day (E) 18.5, *PRMT7*^*−/−*^ lungs were indistinguishable from controls (Fig. 1C), at P2 *PRMT7*^*−/−*^ lungs failed to initiate alveolarization, secondary septa were less frequent, and distal airspaces were enlarged compared with controls, and the more apparent failure of septa formation was detected by P6 in *PRMT7*^−*/*−^ lungs (Fig. 1C). Detailed analyses of inflated lung structures revealed that the average size of alveolar was significantly increased in *PRMT7*^*−/−*^ lungs compared with control mice (Fig. 1D). The mean linear intercept, which describes the mean free distance in airspaces and a surrogate parameter for alveoli, was significantly larger in *PRMT7*^*−/−*^ than in control lungs (Fig. 1E). Collectively, these findings suggest PRMT7 is required for septa formation during lung alveologenesis in mice.

### Decreased number of AMYFs and compromised elastin deposition in postnatal *PRMT7*^*−/*−^ lungs

**Fig. 2:**
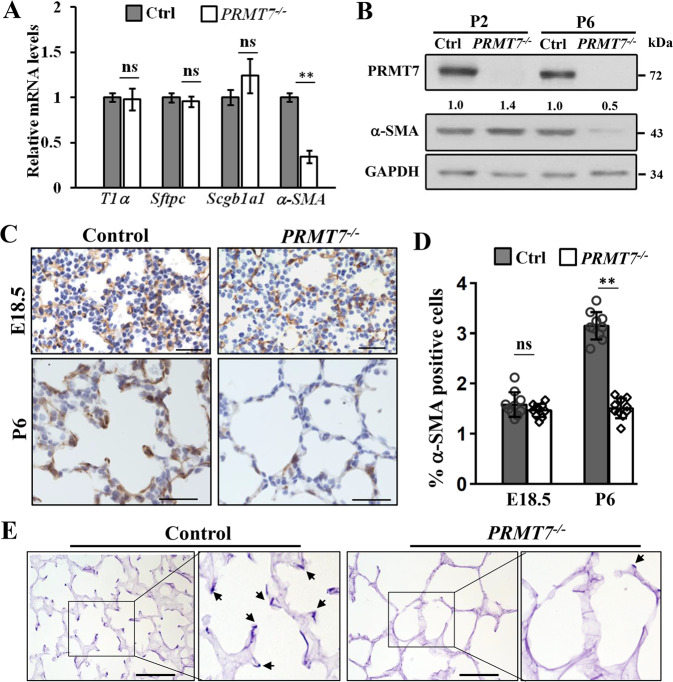
Impaired α-SMA expression and reduced elastin deposition in *PRMT7*^*−/−*^ lungs. **A** qRT-PCR validation of expression of indicated genes in control and *PRMT7*^*−/−*^ lungs at P6. Data were normalized to β-actin and then to the control expression level. *n* = 6 biological replicates. Data were presented as mean ± SD. ns not significant, **P < 0.01 (Student’s *t*-test). **B** PRMT7 and α-SMA protein expression were detected by western blotting in the lung tissues of *PRMT7*^−*/*−^ and control mice at P2 and P6. The relative level of α-SMA are listed above. **C** Representative α-SMA immunostaining images of *PRMT7*^−*/*−^ and control lungs at E18.5 and P6. Scale bars: 100 μm. **D** Quantitative analysis of α-SMA positive cells in the alveolar region. Fifteen fields from at least three mice were calculated for each condition. ns not significant, **P < 0.01 (Student’s *t*-test). **E** Gomori’s aldehyde fuchsin staining of elastin from control and *PRMT7*^*−/−*^ lungs at P6. Magnified pictures from the boxed area were shown on the right panel. Arrows point to positive elastin staining signals. Scale bars: 100 μm.

To investigate the mechanism of the alveologenesis defects in *PRMT7-*knockout mice, we examined cell differentiation in *PRMT7*^*−/−*^ lung tissues by immunofluorescence staining. All checked proximal airway epithelial cell types, including proximal progenitor cells (Sox2^+^), Clara cells (CC10^+^), ciliated cells (Ac-tubulin^+^), and neuroendocrine cells (CGRP^+^), were present in *PRMT7*^−*/−*^ lungs at age of P6 (Fig. S1A). Alveolar type I (ATI) 5 lung epithelial cells (Aqp5^+^, RAGE^+^), and alveolar type II (ATII) lung epithelial cells (Sftpc^+^, Abca3^+^) in lung distal airway also shown corresponding expression and localization in *PRMT7*^*−/*−^ lungs (Fig. S1A). qRT-PCR results showed that T1α (ATI cell marker), Sftpc (ATII cell marker), and Scgb1a1 (Clara cell marker) expression were comparable between control and *PRMT7*-deficient lungs (Fig. 2A). However, one of the mesenchyme cell markers, alpha-smooth muscle actin (α-SMA), which labels parabronchial smooth muscle cells and AMYFs in lung tissues [26], was significantly reduced expressed in *PRMT7*^*−/*−^ mice at P6 (Fig. 2A, B). Immunohistology analysis revealed that the α-SMA^+^ AMYFs from control animals exhibited prominent α-SMA immune reactivity near tips of secondary septa, while the α-SMA immune reactivity was strongly reduced and α-SMA^+^ cells were rarely associated with recognizable secondary septa in *PRMT7* mutant lungs at P6 (Fig. 2C). Moreover, no apparent differences of α-SMA expression were observed between control and *PRMT7*^*−/*−^ lungs at E18.5 (Fig. 2C), and the α-SMA^+^ parabronchial smooth muscle cells and vessel associated α-SMA^+^ cells were also not significantly affected by *PRMT7* deletion at P6 (Fig. S1B). Quantification of α-SMA^+^ cells in the alveolar region revealed a significantly decreased number of AMYFs in *PRMT7*^*−/−*^ lungs at P6 (Fig. 2D). Given that the most important role of mature AMYFs during alveolar morphogenesis is product elastin during alveolar septa formation [5], we conducted Gomori’s aldehyde fuchsin staining for elastin. Enriched elastin was found at the tips of secondary septa in control lungs at P6, whereas the elastin was detected as a disorganized zone rather than foci in the lungs of *PRMT7*^*−/*−^ mice (Fig. 2E). Collectively, these results revealed a crucial role of PRMT7 for the elastin fiber deposit of AMYFs during lung alveolarization.

### Inactivation of *PRMT7* results in decreased AMYFs proliferation

**Fig. 3:**
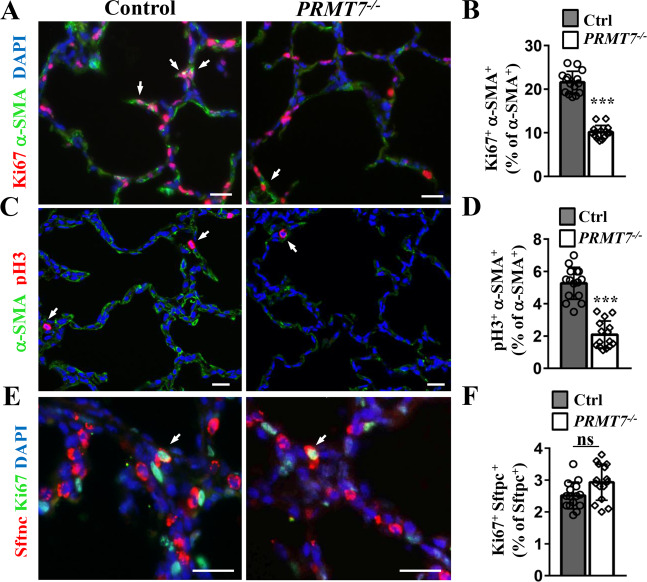
Reduced proliferation of lung alveolar myofibroblasts in *PRMT7*^*−/*−^ mice. **A**, **B** Ki67 and α-SMA co-immunostaining showing the reduced myofibroblasts proliferation in *PRMT7*^*−/*−^ lungs. Arrows indicate Ki67 and α-SMA double-positive cells. Scale bars: 20 μm. proliferating myofibroblasts were quantified as in (**B**). **C** Proliferating myofibroblasts were detected by α-SMA and pH3 co-immunostaining in control and *PRMT7*^*−/−*^ lungs at P6. Arrows indicate pH3 and α-SMA double-positive cells. Scale bars: 20 μm. **D** Quantification of α-SMA and pH3 double-positive cells. **E**, **F** Ki67 and Sftpc co-immunofluorescence staining showing normal alveolar type II (ATII) cell proliferation in *PRMT7*^*−/*−^ lungs. Arrows indicate Ki67 and Sftpc double-positive cells. Scale bars: 20 μm. Proliferating ATII cells were quantified as in (**F**). **B**, **D**, **F** Only the number of α-SMA^+^ cells located within the alveolar region were calculated (excluding parabronchial and vessel associated α-SMA^+^ cells). Data were shown as mean ± SD. Fifteen fields from at least six mice per genotype were quantified. ns not significant, ***P < 0.001 (Student’s *t*-test).

During normal alveolarization, interstitial AMYFs markedly proliferates coinciding with intense alveolar formation [27]. The absence of AMYFs are associated with a lack of secondary septation and alveolarization failure as observed in *PRMT7*-knockout mice. Therefore, we investigate whether proliferation decrease or apoptosis increase in the *PRMT7*-deficient mice. Terminal -deoxynucleotidyl transferase-mediated nick end labeling (TUNEL) analysis indicated that only very few cell deaths occurred in *PRMT7*-deficient lungs as in control (Fig. S2A, B), implying the observed reduction in AMYFs number was not likely caused by apoptosis. Next, we analyzed the AMYFs proliferation status in *PRMT7*-deficient mice. Immunofluorescence co-staining of Ki67/pH3 and α-SMA was performed in lung tissues of control and *PRMT7*-knockout mice at P6. As shown in Fig. 3A–D, both Ki67 and pH3 positive AMYFs were decreased in *PMRT7*^*−/−*^ lungs. Quantitative analysis showed a significant decrease in the number of proliferating α-SMA/Ki67 and α-SMA/pH3 double-labeled cells in the alveolar region of *PRMT7*^*−/−*^ lungs (Fig. 3B, D), while the number of proliferating Ki67/Sftpc double-positive ATII epithelial cells remains comparable between control and *PRMT7*^*−/*−^ lungs (Fig. 3E, F). The decreased AMYF proliferation in *PRMT7*-deficient lungs was further confirmed by using isolated MYFs from the distal lungs. Immunofluorescence staining results showing decreased α-SMA expression in isolated *PRMT7*^*−/−*^ cells, recapitulated the in vivo phenotype of *PRMT7*^−*/−*^ mice (Fig. S2C). Of note, we found expression of PRMT7 was higher in isolated MYFs than in lung tissues (Fig. S2D). Analysis of the proliferation rates by Ki67 and pH3 immunostaining shown a significantly decreased number of both Ki67^+^ and pH3^+^ cells in *PRMT7*^−*/*−^ MYFs than in controls (Fig. S2E–H). Therefore, PRMT7 is required for intestinal MYFs proliferation during lung alveologenesis.

### Impaired AMYFs differentiation in *PRMT7*-deficient lungs

**Fig. 4:**
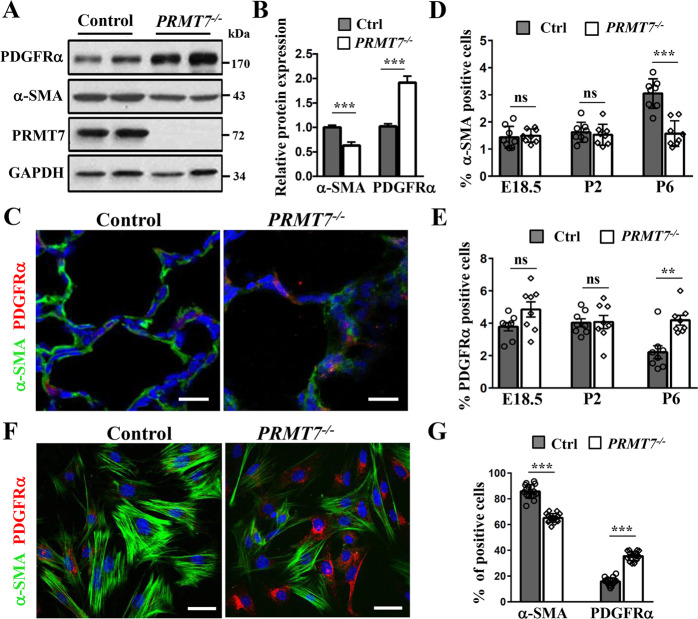
Impaired fibroblast to myofibroblast differentiation in *PRMT7*^*−/−*^ lungs. **A** Western blotting analysis showing the increased PDGFRα and decreased α-SMA expression levels in *PRMT7*^*−/−*^ lungs at P6. **B** Quantitative analysis of relative PDGFRα and α-SMA expression levels. The protein expression levels were normalized to GAPDH and the value for control protein expression level being set as 1. *n* = 4 biological replicates from three independent experiments. ***P < 0.001 (Student’s *t*-test). **C** Representative immunofluorescence images showing fewer α-SMA^+^ cells and more PDGFRα^+^ cells in *PRMT7*^*−/*−^ lungs. Scale bars: 50 μm. **D**, **E** Quantitative analysis of α-SMA^+^ (**D**) and PDGFRα^+^ (**E**) cells in control and *PRMT7*^*−/−*^ lungs at E18.5, P2, and P6. Only the number of α-SMA^+^ or PDGFRα^+^ cells located within the alveolar region were calculated. Data were presented as mean ± SD. *n* = 8 mice for each phenotype and developmental stage. ns not significant,**P < 0.01, ***P < 0.001 (one-way ANOVA with Sidak’s test). **F** Representative double-immunofluorescence staining images of isolated myofibroblasts (MYFs) by using α-SMA and PDGFRα antibodies. Scale bars: 20 μm. **G** Quantitative analysis of α-SMA^+^ and PDGFRα^+^ cells in MYFs. *n* = 15 fields. ***P < 0.001 (one-way ANOVA with Sidak’s test).

Given that the α-SMA positive AMYFs are derived from a population of mesenchymal progenitors expressing PDGFRα [8], we then examined whether PDGFRα expression was affected by *PRMT7* deletion. Western blotting analysis showed a significantly decreased α-SMA and elevated PDGFRα expression levels in *PRMT7*^*−/*−^ lungs at P6 (Fig. 4A, B). Co-immunostaining of α-SMA and PDGFRα also showed a reduced α-SMA and increased PDGFRα expression in *PRMT7*^−*/*−^ lungs at P6 (Fig. 4C). Quantitative analysis results revealed a significantly reduced number of α-SMA^+^ and increased number of PDGFRα^+^ cells at P6, whereas the number of α-SMA^+^ and PDGFRα^+^ cells were comparable between control and *PRMT7*-deficient mice at E18.5 and P2 (Fig. 4D, E). In isolated *PRMT7*^*−/−*^ MYFs, we consistently detected a significantly reduced number of α-SMA^+^ and an increased number of PDGFRα^+^ cells (Fig. 4F, G). Taken together, these results implying that PRMT7 is required for AMYFs differentiation from PDGFRα^+^ precursors to α-SMA^+^ MYFs.

### PRMT7 directly binds to the promoter of *Foxm1* to activate its expression

**Fig. 5:**
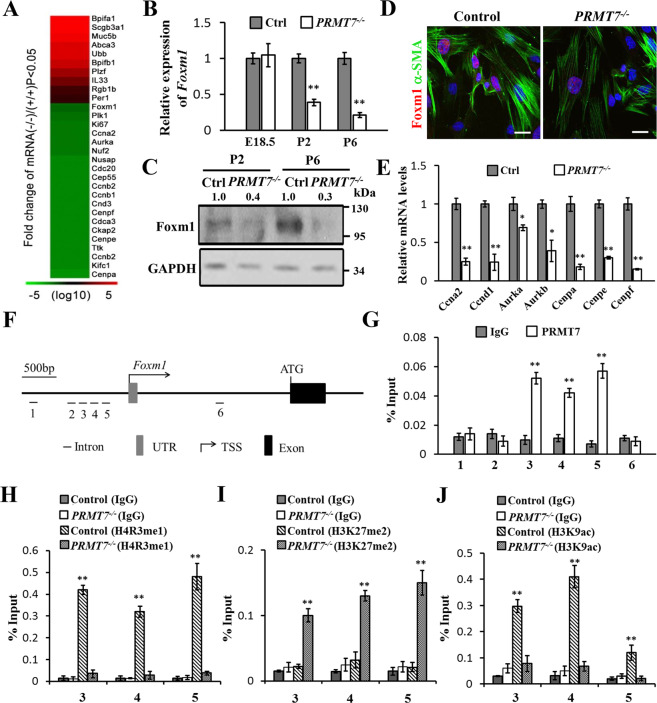
Epigenetic activating of *Foxm1* by PRMT7. **A** RNA-sequencing analysis showing representative genes that were differentially expressed between *PRMT7*^−*/*−^ and control lung tissues. Red, upregulated genes; green, downregulated genes. **B** qRT-PCR analysis to validate the expression of *Foxm1* in *PRMT7*^*−/−*^ and control lung tissues at E18.5, P2, and P6. Gene expression levels were normalized to β-actin. Data were presented as mean ± SD. *n* = 5 biological replicates, **P < 0.01 (Student’s *t*-test). **C** Western blot analysis showing the decreased Foxm1 expression in *PRMT7*^*−/−*^ lungs at P2 and P6; the relative Foxm1 levels are listed above. GAPDH was used as a loading control. The experiment was repeated three times with similar results. **D** Representative immunostaining images showing the decreased Foxm1 expression in isolated *PRMT7*^*−/*−^ MYFs. Scale bars: 20 μm. **E** qRT-PCR validation of expression for levels of *Foxm1* downstream genes. Gene expression levels were normalized to β-actin. Data were presented as mean ± SD. *n* = 6 biological replicates, *P < 0.05, **P < 0.01 (Student’s *t*-test). **F** Schematic diagrams of the mouse *Foxm1* gene structure, with bars representing the regions examined by ChIP, white boxes represent exons; black lines represent introns. **G**–**J** ChIP assay performed with anti-PRMT7 (**G**), anti-H4R3me1 (**H**), anti-H3K27me2 (**I**), and anti-H3K9ac (**J**) antibodies, at indicated regions of the *Foxm1* gene locus in control and *PRMT7*^*−/*−^ lung tissues at P2, *n* = 5 animals for each group. All changes were normalized to inputs. The data shown are the mean ± SEM of three independent experiments. **P < 0.01 (Student’s *t*-test).

To investigate the molecular mechanism involving in AMYFs proliferation and differentiation defects in *PRMT7*^*−/−*^ mice, we conducted RNA-sequencing analysis using total RNA from control and *PRMT7*^*−/*−^ mice at P2. We found a total of 137 gene expression alterations (24 upregulated and 113 downregulated) with a fold change >2 (*p* value <0.05) in *PRMT7*^−*/−*^ lung tissues (Table S1). To reveal whether particular gene classes were enriched among the differentially expressed genes (DEGs), we performed gene ontology (GO) terms related to cellular component using DAVID software, and found that genes associated with cell division, cell cycle, and DNA replication, were significant highly ranked among the top five categories (Fig. S3). Specifically, we found one set of cell cycle and cell proliferation regulating genes, including the proliferation-specific transcription factor *Foxm1* (a typical proliferation factor that plays an important role in organ morphogenesis and development of cancer), and its target genes, such as *Ccnb1*, *Ccnd1*, *Ki67*, *Cenpa*, *Cenpe*, *Cenpf*, *Aurka*, *Plk*1, and *Ccna2*, were significantly decreased in *PRMT7*^*−/*−^ lungs (Fig. 5A). The decreased expression of *Foxm1* in *PRMT7*^*−/*−^ lung tissues was further confirmed by qRT-PCR (Fig. 5B) and western blotting (Fig. 5C). Immunoflourance staining showed that Foxm1 expression was also decreased in isolated MYFs (Fig. 5D). Consistently, significantly decreased expression of *Foxm1* target genes were detected in *PRMT7*^*−/*−^ lung tissues by qRT-PCR at P6 (Fig. 5E). We further determined whether *Foxm1* is a direct target of PRMT7 by chromatin immunoprecipitation (ChIP) assays using chromatin extracts from P2 lungs. With PRMT7 antibody and multiple primer sets within 2 kb region upstream of the mouse *Foxm1* transcriptional start site (TSS), we detected appreciable signals at three sites (3, 4, 5) with a region between 416 and 958 bp upstream of TSS. In contrast, PRMT7 binding with *Foxm1* was not observed in an unrelated intergenic region and the other two detected regions upstream of the *Foxm1* locus (Fig. 5F, G). H4R3me1 binding with *Foxm1* was also highly enriched at the site of 3, 4, and 5 in control lungs, which was significantly reduced in *PRMT7*^*−/*−^ lungs (Fig. 5H), demonstrating specificity. Furthermore, in consistent with the downregulated expression of *Foxm1* in *PRMT7*^*−/−*^ lungs, we observed a significantly increased binding of H3K27me2 (a transcriptional inhibition histone modification), and significantly decreased binding of H3K9ac (a transcriptional activation histone modification) in the *Foxm1* promoter regions of PRMT7 binding with (Fig. 5I, J). However, although the PRMT7 was enriched in chromatin fraction of lung tissues (Fig. S4A), the global level of H4R3me1, as well as other reported histone arginine methylations, such as H4R3mes2, H3R2me2s, H3R17me2s, H3R8me2s, and H3R8mm, were not significantly altered in *PRMT7*^*−/*−^ lungs detected by western blotting (Fig. S4B–E). Thus, the results of these experiments suggest that PRMT7 and H4R3mm1 directly bind with *Foxm1* and positively regulate its transcription, thereby modulating the downstream target genes to control AMYFs proliferation and differentiation.

### Overexpression of *Foxm1* rescues the proliferation and differentiation defects in *PRMT7*-deficient MYFs

**Fig. 6:**
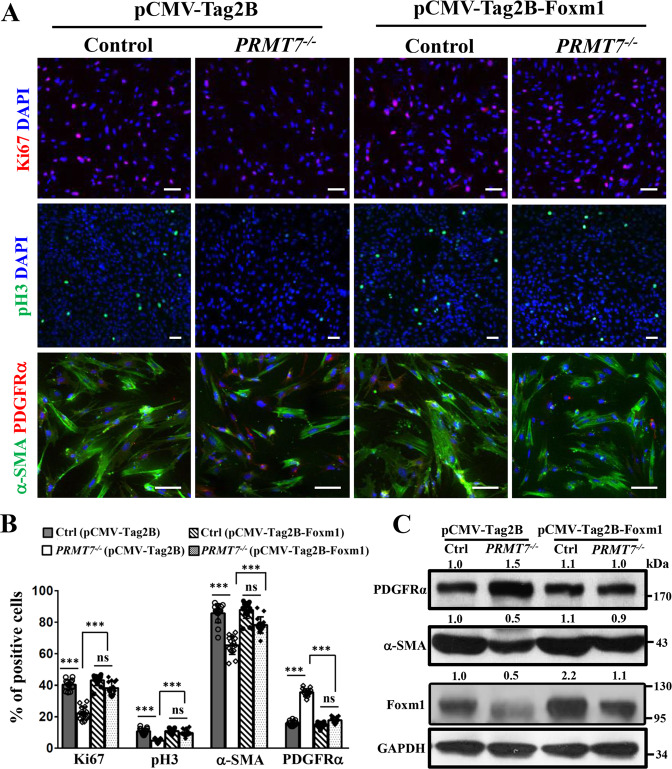
Overexpression of *Foxm1* rescues the proliferation and differentiation defects in *PRMT7*^*−/−*^ myofibroblasts. **A** Representative immunostaining images for Ki67, pH3, α-SMA, and PDGFRα in control and *PRMT7*^*−/−*^ MYFs which were transfected with pCMV-Tag2B or pCMV-Tag2B-Foxm1. Note that both proliferation and differentiation were significantly rescued by Foxm1 overexpression in *PRMT7*-deficient MYFs. Scale bars: 20 μm. **B** Quantification of the number of Ki67^+^, pH3^+^, α-SMA^+^, and PDGFRα^+^ cells. Data were presented as mean ± SD. *n* = 15 fields from six biological replicates of three independent experiments. ns not significant, ***P < 0.001 (one-way ANOVA with Tukey’s test). **C** Western blots analysis showing elevated α-SMA and decreased PDGFRα expression after *Foxm1* overexpression in cultured *PRMT7*^−*/−*^ MYFs. The relative protein expression levels are listed above. GAPDH was used as a loading control. The experiment was repeated three times with similar results.

Given that the previously reported function of Foxm1 during MYFs proliferation and differentiation [28], we further determined whether the decreased Foxm1 expression in *PRMT7* mutant contributes to the lung defects described above. We overexpressed *Foxm1* in isolated MYFs, and MYFs isolated from *PRMT7* mutant lungs exhibited significantly decreased proliferation (as evidenced by immunofluorescence staining of Ki67 and pH3) and differentiation (as evidenced by co-immunofluorescence staining of α-SMA and PDGFRα) phenotypes (Fig. 6A, B), recapitulating the in vivo *PRMT7*-deficient lung phenotypes, suggesting that this in vitro cell culture system would allow us to examine how *Foxm1* expression levels contribute to the defects in *PRMT7*^−*/−*^ lungs. Furthermore, expression of *Foxm1* to the control level significantly rescues both the proliferation and differentiation defects in *PRMT7*-deficient MYFs (Fig. 6A–C), suggesting that the decreased *Foxm1* expression significantly contributes to the lung AMYFs proliferation and differentiation defects in *PRMT7*^*−/−*^ mice.

### Mesenchymal-specific deletion of *PRMT7* mimics the alveolarization defects in *PRMT7*^−*/−*^ mice

**Fig. 7:**
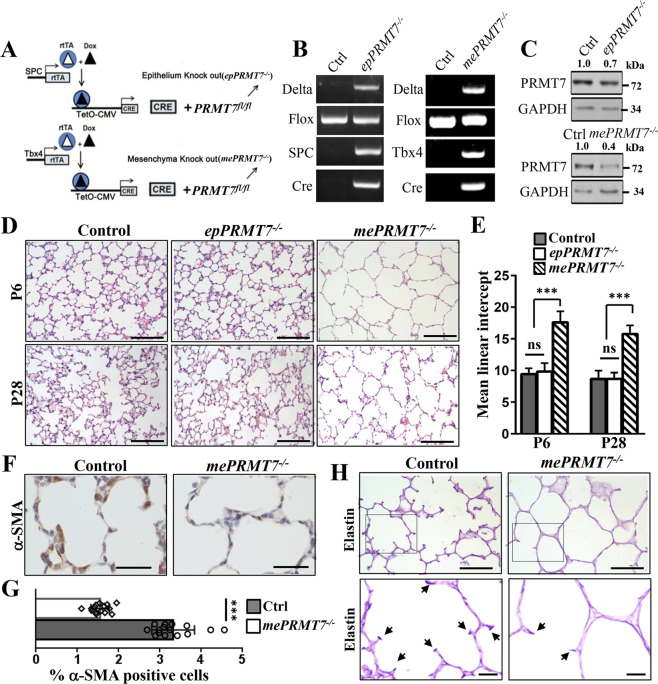
Conditional knockout of *PRMT7* in lung mesenchyme recapture the global *PRMT7*-knockout phenotypes. **A** Schematic of the specifically knockout strategy used to delete the *PRMT7* gene in lung mesenchyme or epithelium lineages. In triple-transgenic mice (*epPRMT7*^−*/*−^ or *mePRMT7*^−*/*−^), rtTA is expressed in epithelium or mesenchyme under the control of SPC or Tbx4 promoter, respectively. In the presence of doxycycline (Dox), rtTA binds to the tetO-CMV promoter to activate Cre-recombinase expression, resulted in recombination and deletion of *PRMT7* gene in *PRMT7*^*fl/fl*^ mice. **B** PCR genotyping of the offspring from epithelium *PRMT7*-knockout (*epPRMT7*^−/−^) and mesenchyme *PRMT7*-knockout (*mePRMT7*^−/−^). **C** Western blotting showing downregulated PRMT7 expression levels in *epPRMT7*^*−/−*^ and *mePRMT7*^*−/−*^ lung tissues. The relative PRMT7 expression level was listed above. GAPDH was served as a loading control. **D** Representative H&E staining images from *epPRMT7*^*−/*−^ and *mePRMT7*^*−/−*^ lung tissues at P6 and P28. Scale bars: 100 μm. **E** Quantitated bar graphs of the mean linear intercept. The mean free distance in airspaces was significantly larger in *mePRMT7*^*−/*−^ lungs at P6 and P28, but no difference between control and *epPRMT7*^*−/*−^ mice. Data were shown as mean ± SD. *n* = 8 mice per genotype. ns not significant, ***P < 0.001 (one-way ANOVA with Tukey’s test). **F** Representative staining images showing decreased α-SMA expression in lung tissues of *mePRMT7*^*−/*−^ at P6. Scale bars: 50 μm. **G** the quantification result of α-SMA positive cells as shown in (**F**). The α-SMA^+^ cells located in alveolar regions were quantified. Data were presented as mean ± SD. *n* = 15 fields from six mice per genotype. ***P < 0.001 (Student’s *t*-test). **H** Representative elastin staining images from lung tissue of control and *mePRMT7*^*−/−*^ mice. Scale bars: 50 μm. The boxed area was magnified below. Arrows indicate a positive elastin signal.

By examining the cellular localization of PRMT7 in lung tissues, we found that PRMT7 is ubiquitously expressed both in lung epithelium and mesenchyme (Fig. S5). To address whether epithelial- or mesenchymal-expressed PRMT7 contributes to the lung developmental defects in *PRMT7*-deficient mice, we conditionally inactivated *the PRMT7* gene in either lung epithelium or mesenchyme using a previously reported inducible gene knockout approach [29, 30] (Fig. 7A). *PRMT7*^*fl/fl*^ mice were bred into TetO-Cre and SPC-rtTA/Tbx4-rtTA mice to produce triple-transgenic mice, in which *PRMT7* was selectively deleted in respiratory epithelial or mesenchymal cells upon administration of doxycycline (Dox). *PRMT7*^*fl/fl*^*;SPC-rtTA;TetO-Cre* (herein referred to as *epPRMT7*^*−/−*^) and *PRMT7*^*fl/fl*^*;Tbx4-rtTA;TetO-Cre* (herein referred to as *mePRMT7*^*−/*−^) triple-transgenic mice were generated by maintaining pregnant dams on Dox from E6.5 onwards. Specifically, Cre expression and the efficiency of Cre-mediated *PRMT7* deletion was examined by PCR and western blotting respectively (Fig. 7B, C). Of note, PRMT7 protein expression was dramatically decreased in *mePRMT7*^*−/*−^ lungs, whereas its expression was slightly decreased in *epPRMT7*^*−/−*^ mice compared with controls (Fig. 7C), implying a higher expression of PRMT7 protein in lung mesenchyme than epithelium. By histology analysis of lung structures from *epPRMT7*^*−/−*^ and *mePRMT7*^−*/*−^ mice, we observed normal alveolar morphogenesis in the lung of *epPRMT7*^*−/*−^ mice at P6 and P28 (Fig. 7D, E). However, mesenchymal-specific deletion of *PRMT7* in lung tissues resulted in failure of septa formation, decreased α-SMA expression, and reduced elastin deposition (Fig. 7D–H), recapture the global *PRMT7*-knockout lung phenotypes. Therefore, these results demonstrate that the mesenchymal-expressed PRMT7 is predominantly required during lung alveologenesis.

## Discussion

**Fig. 8:**
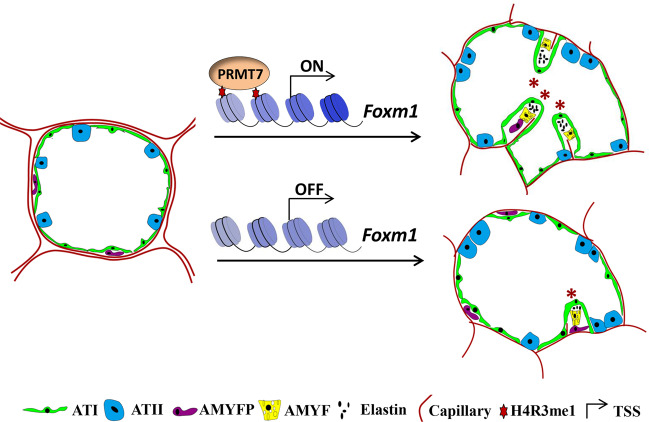
A proposed working model of PRMT7 during lung alveolarization. During lung alveolar morphogenesis, PRMT7 and H4R3me1 directly bind to the promoter of *Foxm1* to activate its expression, and thereby promote the proliferation and differentiation of AMYFs. In the absence of *PRMT7* and H4R3me1, repressed *Foxm1* results in reduced proliferation and differentiation of AMYFs, compromised elastin deposition, failure of septa formation, and eventually form a lung with enlarged distal airspaces structures. The red star indicates the secondary septa.

In this study, we provide direct evidence that protein arginine methyltransferase PRMT7 is required during lung alveologenesis through activation of *Foxm1*. We propose that decreased Foxm1 expression in *PRMT7*-deficient mice resulting in reduced AMYFs proliferation and differentiation, which contributes to impaired elastin deposition and failure of septa formation in *PRMT7*^−*/−*^ lungs (Fig. 8). Our results thus revealed an intriguing epigenetic mechanism whereby PRMT7-mediated H4R3me1 transcriptionally activates *Foxm1* expression to regulate proliferation and differentiation of AMYFs during lung alveologenesis.

Our results reveal an important role for histone methylation during lung alveologenesis. Abnormal alveolarization has devastating effects and is present in human disease conditions such as emphysema or BPD that are characterized by destruction or failure of alveoli development [31]. Previous studies suggested that epigenetic mechanisms, such as histone acetylation, play an important role in regulating lung development. Hdac1/2 is required for proximal epithelium development, loss of which leads to defects in early lung branching morphogenesis [32]. Postnatally, Hdac1/2 was necessary for regeneration but not homeostasis of proximal airway epithelial [33]. The Hdac3-mediated epigenetic pathway is critical for the proper remodeling and expansion of the distal lung saccules into primitive alveoli, epithelial loss of *Hdac3* in lung epithelium leads to a reduction of ATI cell spreading and a disruption of lung alveolarization [34]. However, the physiological effect of histone methylation during lung development remains largely unknown. The present study provided direct evidence that histone arginine methylation writer PRMT7 is essential for lung alveolar morphogenesis. Global or mesenchymal-specific deletion of *PRMT7* gene in mice resulted in failure of septa formation and reduced elastin deposition. Consistently with the function of PRMT7 during lung alveolarization, a significantly highly expressed PRMT7 was detected in lung tissues during P2 to P14, a time period when lungs underwent alveolar morphogenesis and secondary septa formation [2].

Our results identify a typical transcription factor *Foxm1*, as the key target gene of PRMT7 to regulate AMYFs proliferation and differentiation during alveologenesis. Foxm1 is best recognized as a master regulator of cell cycle progression on the basis of studies in a variety of different cell types [28, 35–37], and it is also required for the differentiation and proliferation of many cell lineages during embryonic development [38–41]. Fibroblast-specific deletion of *Foxm1* inhibits fibroblast proliferation, MYF differentiation and therefore protects mice from bleomycin-induced fibrosis [28]. Mice with global deletion of the *Foxm1* gene (*Foxm1*^−*/−*^) exhibited an embryonic lethal phenotype between E13.5 and E16.5, due to structural abnormalities in various organs including the lung [38, 39, 42]. *Foxm1*^*−/−*^ lungs exhibit a significant reduction in proliferation and differentiation of mesenchymal cells during the canalicular stage of lung development, yet proliferation levels were normal in the *Foxm1*-deficient lung epithelial cells [39]. In consistent with this, deletion of *Foxm1* in developing respiratory epithelium using the surfactant-associated protein C (SPC) promoter also did not alter proliferation rates of the epithelium [43]. In support of the specific role of Foxm1 in lung mesenchymal cell proliferation, we observed a significant decreased proliferation of α-SMA^+^ AMYFs and normal proliferation of Sftpc^+^ epithelium in *PRMT7*^*−/*−^ lungs. The observation of significant reduction of AMYFs proliferation, differentiation, and septa formation in the *mePRMT7*^*−/−*^ but not in the *epPRMT7*^*−/*−^ lungs, further support the idea of Foxm1 is specifically required for lung mesenchyme but not epithelium proliferation. Moreover, ectopic expression of *Foxm1* was sufficient for rescuing the defective mitotic entry and differentiation in isolated *PRMT7*^−*/*−^ MYFs, consistent with the previous finding of that Foxm1 was required not only for cell proliferation but also for MYFs differentiation [28].

Our findings presented here indicate that PRMT7 was recruited to the *Foxm1* promoter regions to epigenetic change methylation status of H4R3me1, and thereby activates its transcription. It has been shown that PRMT7 regulates gene expression in a methyltransferase activity-dependent manner, we determine whether the methyltransferase activity of PRMT7 is required for the regulation of target gene expression. Firstly, we found *PRMT7* deletion did not alter the global H4R3 methylation levels and other histone methylation directly or indirectly regulated by PRMT7, which is consistent with a previous study that *PRMT7* knockdown had no significant effect on global H4R3 methylation [44, 45]. However, through ChIP-qPCR analysis, we found that *PRMT7* deficiency markedly decreased H4K3me1 at the Foxm1 promoter in lung tissues. Thus, PRMT7 deletion may lead to the decrease of Foxm1 epigenetic regulation.

In conclusion, the results of this study demonstrate that arginine methylation writer PRMT7 is required for the postnatal lung alveolar morphogenesis by regulating AMYFs proliferation and differentiation through activating of *Foxm1*. These discoveries revealed a prominent role of PRMT7 and histone arginine methylation during lung alveologenesis, and provide a new approach for therapeutic intervention of pulmonary AMYFs dysfunction-related diseases.

## Materials and methods

### Animals

*PRMT7*^*fl/fl*^ mice were generated as previously described [17]. *PRMT7*^*+/*−^ mice were obtained by crossing with *ZP3-Cre* mice. *PRMT7*^*−/−*^ mice were obtained by intercrossing of *PRMT7*^*+/−*^mice. *PRMT7*^*+/+*^, *PRMT7*^*+/*−^, and *PRMT7*^*−/*−^ littermates, both male and female between the ages of E18.5, P2, and P6 were used in this study. *PRMT7*^*+/+*^littermates are denoted as control mice. The lung epithelial-specific *PRMT7* knockout mice (*epPRMT7*) were generated by crossing *PRMT7*^*fl/fl*^ mice with *SPC-rtTA* [30] and *TetO-Cre* mice [29]. Mesenchymal conditional knockout mice (*mePRMT7*) were generated by crossing *PRMT7*^*fl/fl*^ mice with *TBX4-rtTA* [46] and *TetO-Cre* mice. *SPC-rtTA and TetO-Cre* transgenic mice were kindly gifted by Prof. Huajing Wan (Sichuan University). *TBX4-rtTA* transgenic mouse line was from Prof. Wen Ning (Nankai University). Administration of Dox started from E6.5 to the endpoint of the experiment by feeding the pregnant mice with 625 mg/kg of Dox-containing food.　All mice used in this study were bred in the C57BL/6 strain background and housed in a specific pathogen-free condition with a 12 h light/12 h dark cycle in a temperature- and humidity-controlled environment. Mice were genotyped by genomic DNA PCR using primers listed in Table S2 and then were randomly assigned to their conditions.

### Histologic analysis, immunohistochemical staining, and H&E staining

For histologic analysis, lungs from control or *PRMT7* mutant mice were fixed in 4% (w/v) paraformaldehyde (PFA, Sigma-Aldrich, USA) overnight at 4 °C. After paraffin (Thermo, USA) embedding, tissues were sectioned at 5 µm. For immunohistochemical staining, sections were deparaffinized and rehydrated. Antigen retrieval was performed using the microwave method [17]. Sections were then pretreated with 0.1% Triton X-100 and blocked with 5% BSA for 2 h, followed by incubation with diluted primary antibodies in 3% BSA overnight at 4 °C, 1 h incubation with selected secondary antibodies, and counterstained with hematoxylin. Antibodies used for histologic analysis were listed in Table S3. H&E staining was performed as previously described [17]. Briefly, sections were deparaffinized and rehydrated, followed by staining in harris hematoxylin solution for 8 min. Then, differentiate in 1% acid alcohol for 30 s and counterstain in eosin-phloxine solution for 1 min. At last, dehydrate, clear, and mount.

### Immunofluorescence staining, imaging, and quantification

Immunofluorescence staining was performed following a previously published protocol [47] with antibodies listed in Table S3. Briefly, fixed tissues were transferred into 30% sucrose (Sigma, S8501) overnight at 4 °C and embedded with Optimal Cutting Temperature Compound (TFM-5, USA). Six-μm-thick sections were pretreated with 0.5% Triton X-100 in PBS for 15 min at room temperature. Then, samples were incubated with primary antibodies in 3% BSA overnight at 4 °C, followed by secondary antibodies and counterstained with DAPI for 1 h at room temperature. Images were acquired with an Olympus microscope system (Olympus, Japan) and analyzed with Photoshop software (Adobe Systems Software Ireland Ltd, USA). For quantification of lung sections, images were obtained from a minimum of four mice per genotype, and at least three different areas were counted per image of each mouse. For quantification of in vitro cultured cells, multiple fields were calculated as indicated in the figure legends. All quantification experiments were repeated at least three times with similar results.

### Gomori’s aldehyde fuchsin staining and TUNEL assay

Gomori’s aldehyde fuchsin staining was performed following g a previously described protocol [48]. Images were obtained from a minimum of three mice per genotype, and a representative image for each mouse was shown. TUNEL assay was performed as described previously [47]. For quantification, images were obtained from six mice per genotype. Cells were counted in at least three different areas of each image for each mouse.

### Lung MYFs isolation, culture, and transfection

Lung MYFs were isolated from control and *PRMT7*^*−/−*^ animals using a previously reported protocol [49]. In brief, the trachea and proximal airway were first removed, and the edge of the remaining distal lungs were cut and collected by manual dissection. Collected tissues were then finely minced using sterile scissors and digested with 2 mL trypsin (Gibco, USA) for 20 min at 37 °C with shaking at 5-min intervals. The resulting cell suspension was added to 2 mL of grown medium (Dulbecco’s modified Eagle’s medium (DMEM) (Invitrogen, 12100-046), supplemented with 10% fetal bovine serum (FBS). Cells were washed twice with grown medium and plated in six-well plates (NEST, China). Isolated MYFs were cultured in a growth medium plus 100 U/mL penicillin/streptomycin (Gbico, USA) at 37 °C with 5% carbon dioxide. Cells were tested for mycoplasma contamination using TaKaRa PCR Mycoplasma Detection Set (Clontech Laboratories, EUA).

For rescue experiments, MYFs at 60–70% confluence were transfected with the pCMV-Tag2B-Foxm1 or empty vector using Lipofectamine^TM^ 2000 (Invitrogen, 11668019) as described previously [50]. In brief, replace the growth medium before 1 h of the transfection, mixed the plasmids and transfection reagents at 1:3 ratios, and incubated for 20 min at room temperature before added to the cells. Six hours later, cells were maintained in a fresh medium for 48 h before being fixed or harvested.

### Chromatin immunoprecipitation (ChIP) and western blotting assay

Approximately ten lungs from control and *PRMT7*^*−/−*^ mice at age of P2 were isolated and pooled in a ChIP assay as previously described [47]. Primers for quantitative PCR detection of *Foxm1* chromatin regions are listed in Table S2. For western blotting, proteins were extracted from lung tissues or cultured MYFs using RIPA buffer (50 mM Tris (pH7.4), 150 mM NaCl, 0.5% sodium deoxycholate, 0.1% SDS, and 1% NP-40) contains proteinase inhibitors (Sigma, P8340). Protein concentration was determined using the BCA assay as previously reported [51]. About 20 μg of total proteins were subjected to a standard western blot analysis using a protocol as previously reported [52]. The antibodies used were listed in Table S3.

### RNA extraction, reverse transcription, and quantitative real-time PCR (qRT-PCR)

RNA was extracted using TRIZOL reagent (Ambion, USA) and reversed transcribed with a FastQuant RT Kit (TIANGEN, China) according to the manufacturer’s protocol. RNA was normalized and used for an Agilent Technologies StrataGene Mx3000P real-time PCR system (Agilent, USA). The transcription levels were normalized to internal β-actin expression. At least three biological replicates were performed per genotype and three technical replicates were performed for every independent experiment.

### RNA-sequencing assays

For RNA-sequencing, total RNA was extracted from control and *PRMT7*^*−/−*^ lungs at P2. For each sample, five lungs were collected and pooled. Sequencing libraries were generated using NEBNext^®^ Ultra™ RNA Library Prep Kit (NEB, USA) and sequenced on an Illumina Hiseq platform (Illumina, USA). The 125 bp/150 bp paired-end reads were generated and reads were aligned to the mouse reference genome (mm^10^) using TopHat v2.0.12. HTSeqv0.6.1 was used to count the reads numbers, and then the FPKM of each gene was calculated based on the length of the gene and the reads count mapped to this gene. Differential expression analysis was performed using the DESeqR package (1.18.0).

### Statistical analysis

All experiments were performed using 4–20 mice or at least three independent repeated experiments with cells. The exact sample size (*n*) for each experiment was specified in the figure legends. Animals were not excluded from the analysis. Data were expressed as means ± SD or ±SEM, as indicated in the figure legends. Statistical significance were analyzed by GraphPad Prism version 6 (GraphPad Software, San Diego, CA). A two-tailed unpaired Student’s *t*-test was used for the comparison between two experimental groups. When multiple comparisons were necessary, one-way ANOVA was performed. Prior to making comparisons across values, the normality of distributions was tested and the variance was similar between groups. All statistics are representative of biological replicates. ns, not significant, *P < 0.05, **P < 0.01, ***P < 0.001.

## Supplementary information

Supplemental material

## Data Availability

All data generated or analyzed during this study are included in this published article and its supplementary files.
