# Supplementary material for: PRMT7 targets of Foxm1 controls alveolar myofibroblast proliferation and differentiation during alveologenesis
Source: Cell Death Dis. 2021 Sep 8;12(9):841. doi: 10.1038/s41419-021-04129-1 (PMC8426482; doi:10.1038/s41419-021-04129-1)
Supplement: Supplementary file 1 — Supplemental material [file 41419_2021_4129_MOESM1_ESM.docx]

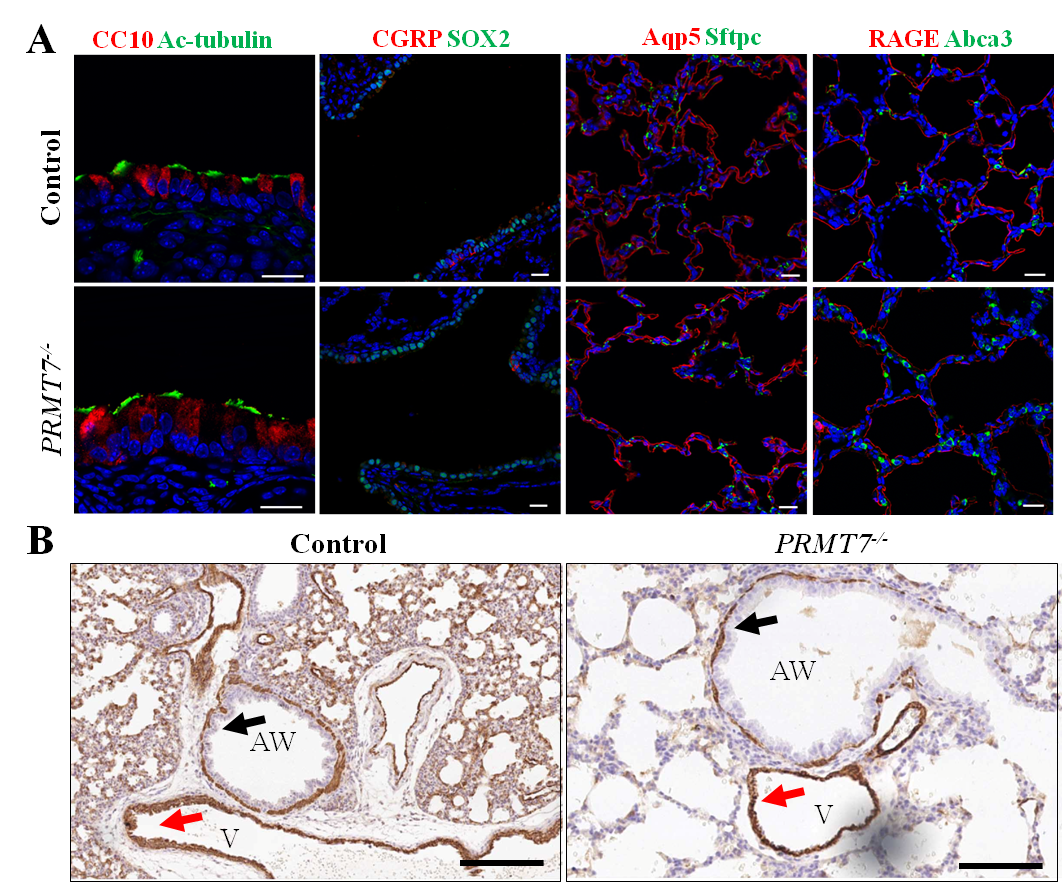


**Fig. S1. Normal cell differentiation in lung epithelium of *PRMT7^-/-^* mice.**

(A) Representative immunostaining images showing that indicated lung epithelial markers are expressed normally in *PRMT7^-/-^* mice at P6. Scale bars: 20 μm. (B) Immunohistochemical staining of α-SMA in control and *PRMT7^-/-^* lungs at P6. Black arrows indicate the SMA^+^ cells around the airway (AW), red arrows indicate vescular (V) SMA^+^ cells. Scale bars: 100 μm.


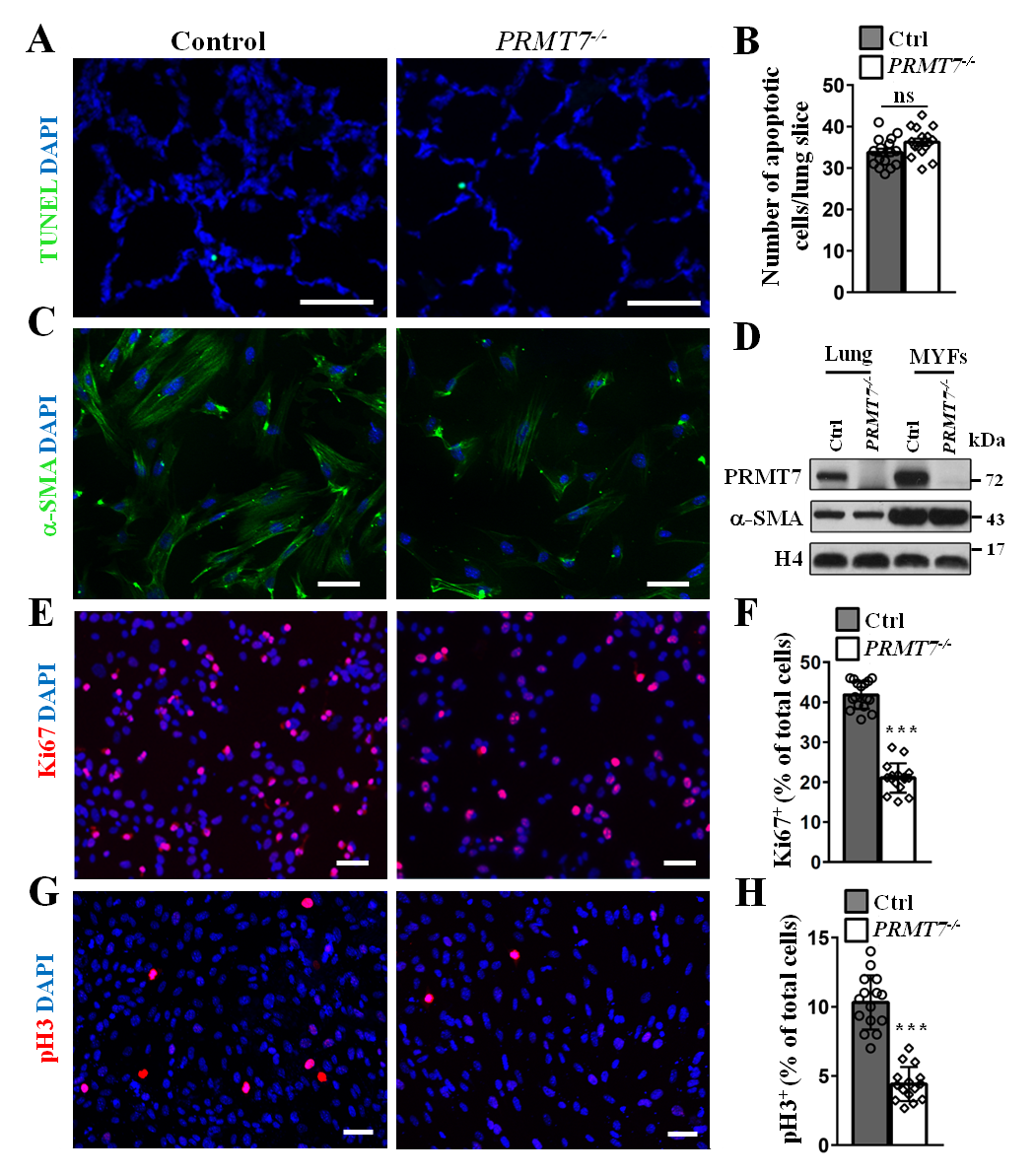


**Fig. S2. Apoptosis and proliferation analysis of alveolar myofibroblasts in *PRMT7^-/-^* lungs.**

(A) TUNEL staining of apoptotic cells in control and *PRMT7^-/-^* lung slices at P6. Scale bars: 50 μm. (B) Quantification of the number of apoptotic cells in control and *PRMT7^-/-^* lungs. Data are presented as mean ± SD. n=6 biological replicates. ns, not significant (Student’s *t*-test). (C) Immunostaining of α-SMA in myofibrolasts (MYFs) isolated from the distal lungs of control and *PRMT7^-/-^* mice. Scale bars: 20 μm. (D) Western blotting analysis of α-SMA expression in lung tissues and isolated MYFs. Note that a remarkable increased α-SMA levels in MYFs than lung tissues. (E-H) Representative immunofluorescence staining image of Ki67 (E) and pH3 (G), and quantification of Ki67^+^ (E) and pH3^+^ (G) cells in cultured MYFs from *PRMT7^-/-^* and control mice. Scale bars: 20 μm. For (F) and (H), Data are presented as mean ± SD. n=9 biological replicates from three independent experiments. ***P<0.001 (Student's *t*-test).


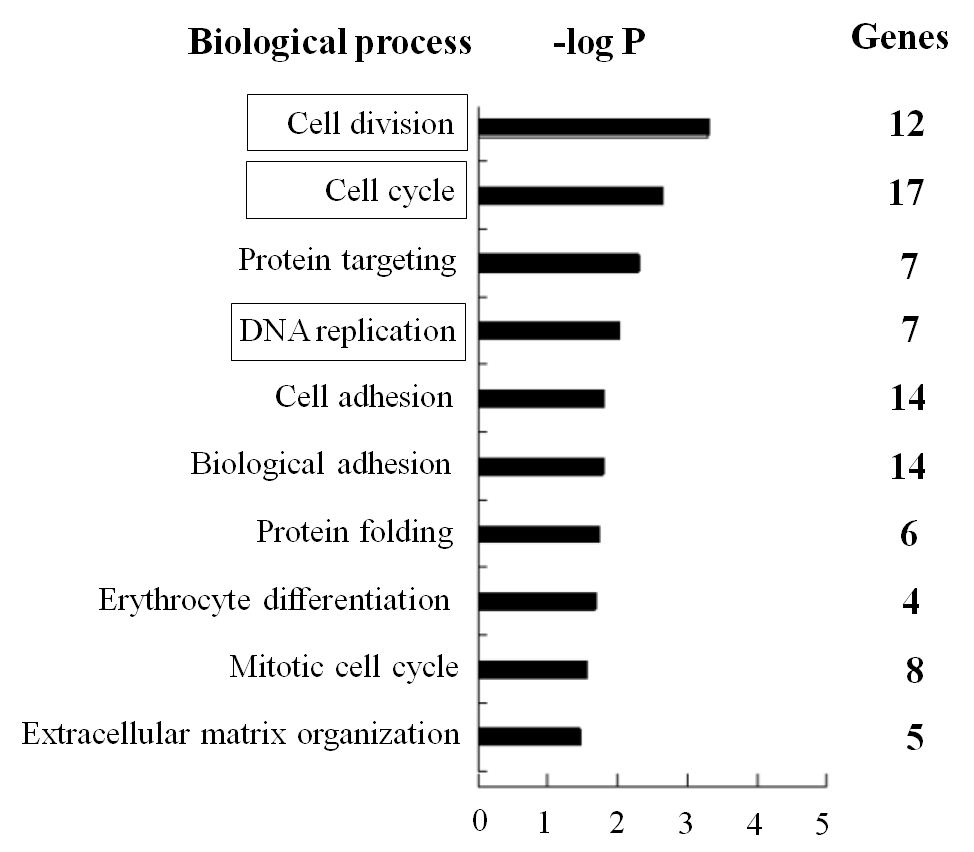


**Fig. S3. Gene ontology (GO) analysis of the differentially expressed genes between control and *PRMT7^-/-^* lungs.**


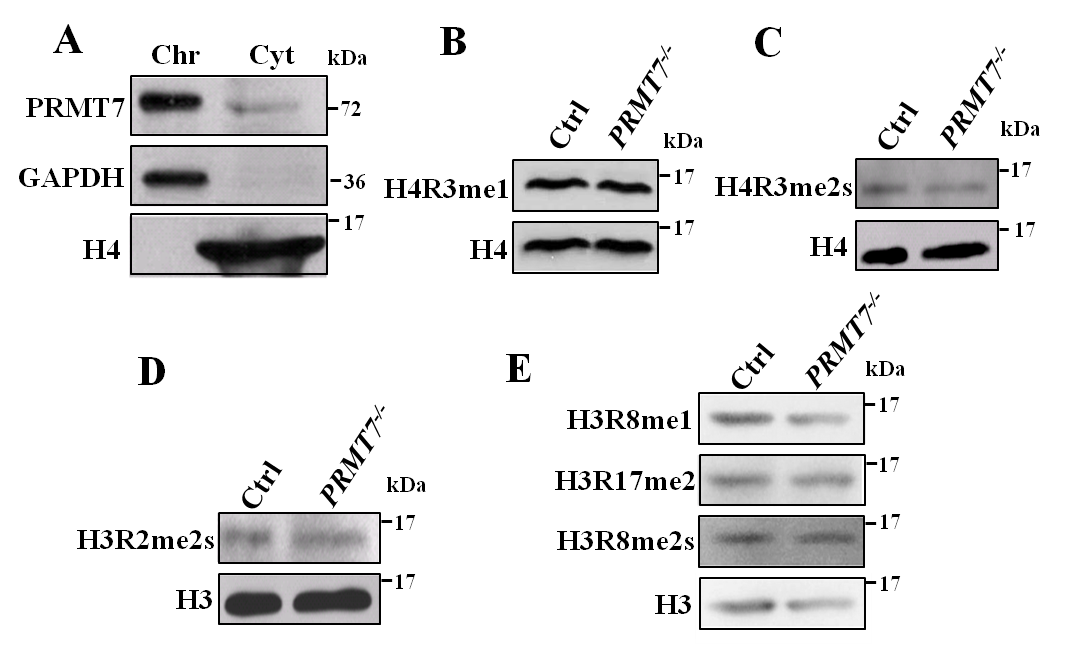


**Fig. S4.** **Detection of histone arginine methylation in *PRMT7^-/-^* lungs.**

(A) Western blot analysis showing the PRMT7 was predominantly expressed in chromatin of lung tissues. Chr: chromatin, Cyt: cytoplasm. (B-E) Western blot analysis the expression levels of different histone modification, such as H4R3me1 (B), H3R2me2s (C), H3R2me2s (D), H3R8me1, H3R17me2 and H3R8me2s (E), from total cell extraction of P2 lung tissues. All experiment were repeated at least three times with similar results.


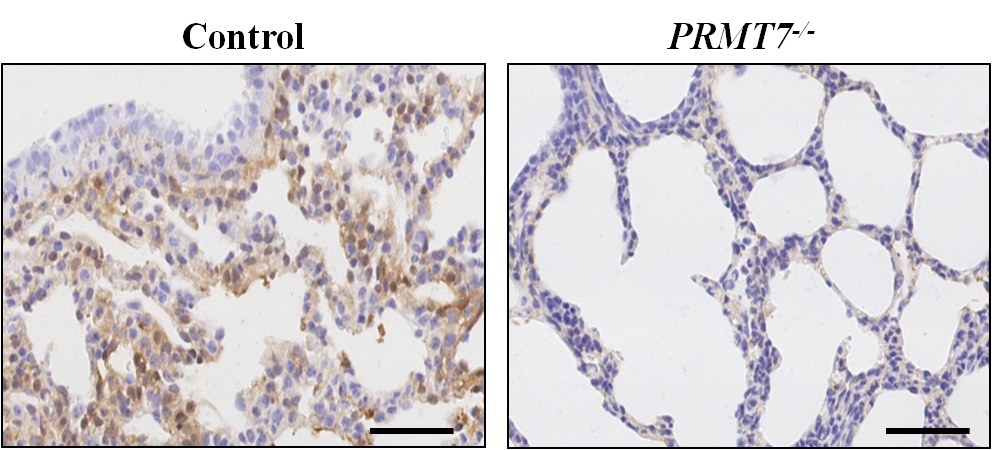


**Fig. S5. The expression pattern of endogenous PRMT7 in lung tissues.**

Representative immunohistochemical staining images showing the endogenous PRMT7 expression in lung tissues at P2. Scale bars: 50 μm.

**Table S1. Differentially expressed genes between control and *PRMT7^-/-^* lungs.**

| **Gene ID** | **Gene** | **Control** | ***PRMT7^-/-^*** |
| --- | --- | --- | --- |
| ENSMUSG00000001228 | Uhrf1 | 28.70137068 | 11.90038951 |
| ENSMUSG00000001403 | Ube2c | 40.73019734 | 7.702895879 |
| ENSMUSG00000001517 | Foxm1 | 48.74265151 | 13.01972274 |
| ENSMUSG00000002055 | Spag5 | 39.59816624 | 6.546095565 |
| ENSMUSG00000003038 | Hmgn2 | 461.2534909 | 208.5241071 |
| ENSMUSG00000003477 | Inmt | 5.003081527 | 31.93711625 |
| ENSMUSG00000003779 | Kif20a | 12.37341276 | 2.649451956 |
| ENSMUSG00000004328 | Hif3a | 10.13731394 | 34.19818998 |
| ENSMUSG00000005233 | SPC25 | 37.71125286 | 8.865067487 |
| ENSMUSG00000006154 | EPS8l1 | 27.61962255 | 64.1542527 |
| ENSMUSG00000006205 | Htra1 | 164.4165392 | 69.05067808 |
| ENSMUSG00000006398 | Cdc20 | 68.39396183 | 12.4412185 |
| ENSMUSG00000006574 | Slc4a1 | 86.95427474 | 25.42117499 |
| ENSMUSG00000007080 | Pole | 20.04837249 | 6.322832306 |
| ENSMUSG00000012443 | Kif11 | 17.47893972 | 3.470419136 |
| ENSMUSG00000015880 | Ncapg | 13.23720934 | 2.356902244 |
| ENSMUSG00000017716 | Birc5 | 22.43400939 | 4.438227096 |
| ENSMUSG00000019942 | Cdk1 | 69.85576812 | 16.71964894 |
| ENSMUSG00000020330 | Hmmr | 19.32739009 | 3.960892804 |
| ENSMUSG00000020493 | Prr11 | 8.870384138 | 1.732327845 |
| ENSMUSG00000020641 | Rsad2 | 20.58012308 | 3.376678225 |
| ENSMUSG00000020649 | Rrm2 | 65.69081661 | 18.01984944 |
| ENSMUSG00000020808 | Fam64a | 59.54905703 | 10.69516665 |
| ENSMUSG00000020897 | Aurora kinase B | 48.3498447 | 11.85109756 |
| ENSMUSG00000020914 | [Top2a](http://asia.ensembl.org/mus_musculus_nzohlltj/Gene/Summary?g=MGP_NZOHlLtJ_G0019513&db=core) | 163.7456845 | 39.69940305 |
| ENSMUSG00000021485 | [Mxd3](http://asia.ensembl.org/mus_musculus_nzohlltj/Gene/Summary?g=MGP_NZOHlLtJ_G0021018&db=core) | 24.68512045 | 3.862965559 |
| ENSMUSG00000022033 | [Pbk](http://asia.ensembl.org/mus_musculus_casteij/Gene/Summary?g=MGP_CASTEiJ_G0020743&db=core) | 38.46929597 | 8.755796789 |
| ENSMUSG00000023015 | [Racgap1](http://asia.ensembl.org/mus_musculus_nzohlltj/Gene/Summary?g=MGP_NZOHlLtJ_G0022761&db=core) | 90.31741927 | 16.88101306 |
| ENSMUSG00000023505 | [Cdca3](http://asia.ensembl.org/mus_musculus_lpj/Gene/Summary?g=MGP_LPJ_G0031169&db=core) | 44.58438932 | 7.810708291 |
| ENSMUSG00000024132 | [Eci1](http://asia.ensembl.org/mus_musculus_cbaj/Gene/Summary?g=MGP_CBAJ_G0022999&db=core) | 23.43022453 | 61.76396526 |
| ENSMUSG00000024590 | Lamin B1 | 111.0557261 | 40.34403107 |
| ENSMUSG00000024610 | CD74 | 89.85305726 | 31.29137822 |
| ENSMUSG00000024660 | [Incenp](http://asia.ensembl.org/mus_musculus_lpj/Gene/Summary?g=MGP_LPJ_G0024754&db=core) | 28.29191181 | 9.046943289 |
| ENSMUSG00000024795 | [Kif20b](http://asia.ensembl.org/mus_musculus_casteij/Gene/Summary?g=MGP_CASTEiJ_G0024267&db=core) | 7.326746592 | 2.186387308 |
| ENSMUSG00000024810 | [Il33](http://asia.ensembl.org/mus_musculus_nzohlltj/Gene/Summary?g=MGP_NZOHlLtJ_G0025501&db=core) | 16.26934814 | 81.38568106 |
| ENSMUSG00000024989 | [Cep55](http://asia.ensembl.org/mus_musculus_nzohlltj/Gene/Summary?g=MGP_NZOHlLtJ_G0025559&db=core) | 13.32706557 | 2.42426276 |
| ENSMUSG00000025574 | [Tk1](http://asia.ensembl.org/mus_musculus_dba2j/Gene/Summary?g=MGP_DBA2J_G0019286&db=core) | 51.4030885 | 17.86852539 |
| ENSMUSG00000026360 | [Rgs2](http://asia.ensembl.org/mus_musculus_nzohlltj/Gene/Summary?g=MGP_NZOHlLtJ_G0017005&db=core) | 88.23611789 | 43.49070827 |
| ENSMUSG00000026390 | [Marco](http://asia.ensembl.org/mus_musculus_cbaj/Gene/Summary?g=MGP_CBAJ_G0016149&db=core) | 4.822126261 | 28.37902702 |
| ENSMUSG00000026605 | [Cenpf](http://asia.ensembl.org/mus_musculus_nzohlltj/Gene/Summary?g=MGP_NZOHlLtJ_G0017365&db=core) | 31.33320368 | 5.521885257 |
| ENSMUSG00000026683 | NUF2 | 18.83519913 | 3.836837684 |
| ENSMUSG00000026822 | Lcn2 | 77.58230079 | 157.7521583 |
| ENSMUSG00000027070 | Lrp2 | 6.239893067 | 17.88657734 |
| ENSMUSG00000027239 | Mdk | 266.8400783 | 117.4500993 |
| ENSMUSG00000027306 | [Nusap1](http://asia.ensembl.org/mus_musculus_casteij/Gene/Summary?g=MGP_CASTEiJ_G0025529&db=core) | 35.2963457 | 6.584325206 |
| ENSMUSG00000027326 | Knl1 | 9.773791758 | 2.005520687 |
| ENSMUSG00000027331 | Knstrn | 40.4303149 | 8.729815511 |
| ENSMUSG00000027379 | [Bub1](http://asia.ensembl.org/mus_musculus/Gene/Summary?g=ENSMUSG00000027379&db=core) | 28.01305716 | 6.66670261 |
| ENSMUSG00000027469 | [Tpx2](http://asia.ensembl.org/mus_musculus/Gene/Summary?g=ENSMUSG00000027469&db=core) | 40.46054694 | 8.406513904 |
| ENSMUSG00000027483 | Bpifa1 | 0.201009475 | 21.31720395 |
| ENSMUSG00000027485 | Bpifb1 | 0.591462057 | 8.615809923 |
| ENSMUSG00000027496 | Aurora kinase A | 24.36008921 | 5.046477989 |
| ENSMUSG00000027715 | Cyclin A2 | 50.3601188 | 10.471622 |
| ENSMUSG00000028068 | Iqgap3 | 15.60479478 | 2.554212428 |
| ENSMUSG00000028128 | F3 | 26.70233013 | 57.16397295 |
| ENSMUSG00000028312 | [Smc2](http://asia.ensembl.org/mus_musculus/Gene/Summary?g=ENSMUSG00000028312&db=core) | 34.35072814 | 11.6482013 |
| ENSMUSG00000028551 | [Cdkn2c](http://asia.ensembl.org/mus_musculus_lpj/Gene/Summary?g=MGP_LPJ_G0028487&db=core) | 37.52663758 | 9.166103177 |
| ENSMUSG00000028678 | Kif2c | 27.90362895 | 5.629925571 |
| ENSMUSG00000028718 | [Stil](http://asia.ensembl.org/mus_musculus_lpj/Gene/Summary?g=MGP_LPJ_G0028507&db=core) | 14.88543468 | 3.909846209 |
| ENSMUSG00000028832 | [Stmn1](http://asia.ensembl.org/mus_musculus_akrj/Gene/Summary?g=MGP_AKRJ_G0028838&db=core) | 405.6925719 | 160.6071709 |
| ENSMUSG00000028873 | Cdca8 | 29.54666498 | 6.534494588 |
| ENSMUSG00000028967 | [Errfi1](http://asia.ensembl.org/mus_musculus_lpj/Gene/Summary?g=MGP_LPJ_G0029059&db=core) | 47.19352896 | 115.2860271 |
| ENSMUSG00000029177 | [Cenpa](http://asia.ensembl.org/mus_musculus_lpj/Gene/Summary?g=MGP_LPJ_G0029294&db=core) | 69.67790836 | 10.18437981 |
| ENSMUSG00000029333 |  | 16.23561274 | 39.92939039 |
| ENSMUSG00000030017 | Reg3g | 0.303622704 | 16.44144553 |
| ENSMUSG00000030677 | Kif22 | 24.70282707 | 4.974514663 |
| ENSMUSG00000030867 | Plk1 | 18.50144861 | 4.165434854 |
| ENSMUSG00000031004 | Mki67 | 45.50275931 | 9.550075432 |
| ENSMUSG00000031636 | [Pdlim3](http://asia.ensembl.org/mus_musculus_lpj/Gene/Summary?g=MGP_LPJ_G0033486&db=core) | 73.91110761 | 34.61617845 |
| ENSMUSG00000032218 | Cyclin B2 | 40.71322349 | 6.73005894 |
| ENSMUSG00000032254 | Kif23 | 21.8961154 | 5.044386561 |
| ENSMUSG00000032425 | Zfp949 | 43.70294197 | 8.493944583 |
| ENSMUSG00000033031 | Cip2a | 20.37388371 | 5.030854992 |
| ENSMUSG00000033952 | [Aspm](http://asia.ensembl.org/mus_musculus/Gene/Summary?g=ENSMUSG00000033952&db=core) | 10.82697711 | 2.174302768 |
| ENSMUSG00000034311 | Kif4 | 18.94058752 | 3.774628312 |
| ENSMUSG00000034906 | [Ncaph](http://asia.ensembl.org/mus_musculus_lpj/Gene/Summary?g=MGP_LPJ_G0026387&db=core) | 50.395947 | 16.50327296 |
| ENSMUSG00000035435 | [Abca17](http://asia.ensembl.org/mus_musculus_nzohlltj/Gene/Summary?g=MGP_NZOHlLtJ_G0023741&db=core) | 0.711940404 | 13.21333253 |
| ENSMUSG00000035683 | Melk | 25.32719388 | 4.307225828 |
| ENSMUSG00000036381 | [P2ry14](http://asia.ensembl.org/mus_musculus/Gene/Summary?g=ENSMUSG00000036381&db=core) | 62.69200363 | 23.67341119 |
| ENSMUSG00000036594 | H2-Aa | 33.41382721 | 5.848557375 |
| ENSMUSG00000036768 | Kif15 | 9.400480504 | 2.672638337 |
| ENSMUSG00000036777 | Anillin | 15.74815241 | 4.076647318 |
| ENSMUSG00000037313 | Tacc3 | 56.83777038 | 12.31891215 |
| ENSMUSG00000037379 | Spon2 | 57.52357432 | 27.7439559 |
| ENSMUSG00000037544 | Dlgap5 | 17.35425453 | 3.714232266 |
| ENSMUSG00000037725 | [Ckap2](http://asia.ensembl.org/mus_musculus/Gene/Summary?g=ENSMUSG00000037725&db=core) | 16.85975668 | 2.94351938 |
| ENSMUSG00000038252 | Ncapd2 | 169.3369011 | 42.44960807 |
| ENSMUSG00000038379 | Ttk | 14.88253137 | 2.47697074 |
| ENSMUSG00000038591 | Colec10 | 2.425523099 | 9.600199392 |
| ENSMUSG00000038943 | [Prc1](http://asia.ensembl.org/mus_musculus/Gene/Summary?g=ENSMUSG00000038943&db=core) | 52.64960801 | 13.81544159 |
| ENSMUSG00000039396 | Neil3 | 31.66234499 | 5.313126066 |
| ENSMUSG00000039934 | Gsap | 11.68055594 | 29.11387584 |
| ENSMUSG00000040084 | Bub1b | 51.19355538 | 12.44512642 |
| ENSMUSG00000040204 | Pclaf | 32.65398925 | 8.838595411 |
| ENSMUSG00000040809 | Chil3 | 16.57237309 | 174.7611423 |
| ENSMUSG00000041064 | Pif1 | 7.505993418 | 0.865716407 |
| ENSMUSG00000041219 | [Arhgap11a](http://asia.ensembl.org/mus_musculus_lpj/Gene/Summary?g=MGP_LPJ_G0026235&db=core) | 20.65162063 | 5.426128805 |
| ENSMUSG00000041431 | [Ccnb1](http://asia.ensembl.org/mus_musculus_cbaj/Gene/Summary?g=MGP_CBAJ_G0020597&db=core) | 34.72006048 | 6.256917665 |
| ENSMUSG00000042029 | [Ncapg2](http://asia.ensembl.org/mus_musculus/Gene/Summary?g=ENSMUSG00000042029&db=core) | 9.549471949 | 3.173718323 |
| ENSMUSG00000042306 | [S100a14](http://asia.ensembl.org/mus_musculus_lpj/Gene/Summary?g=MGP_LPJ_G0027406&db=core) | 35.29053053 | 82.86545411 |
| ENSMUSG00000042489 | [Clspn](http://asia.ensembl.org/mus_musculus_lpj/Gene/Summary?g=MGP_LPJ_G0028695&db=core) | 25.82850666 | 7.098061942 |
| ENSMUSG00000044285 | [Ubb-ps](http://asia.ensembl.org/mus_musculus/Gene/Summary?g=ENSMUSG00000044285&db=core) | 4.483385251 | 82.54951701 |
| ENSMUSG00000045328 | [Cenpe](http://asia.ensembl.org/mus_musculus_lpj/Gene/Summary?g=MGP_LPJ_G0027828&db=core) | 10.23905682 | 1.744391799 |
| ENSMUSG00000047534 | [Mis18bp1](http://asia.ensembl.org/mus_musculus_cbaj/Gene/Summary?g=MGP_CBAJ_G0019494&db=core) | 12.82121555 | 2.49486132 |
| ENSMUSG00000048327 | [Ckap2l](http://asia.ensembl.org/mus_musculus_lpj/Gene/Summary?g=MGP_LPJ_G0026424&db=core) | 16.93746719 | 3.92915786 |
| ENSMUSG00000049932 | [H2ax](http://asia.ensembl.org/mus_musculus/Gene/Summary?g=ENSMUSG00000049932&db=core) | 53.1353443 | 20.24425274 |
| ENSMUSG00000051378 | Kif18b | 16.71501122 | 2.694425852 |
| ENSMUSG00000054717 | Hmgb2 | 129.4114234 | 41.79293146 |
| ENSMUSG00000058290 | [Espl1](http://asia.ensembl.org/mus_musculus_cbaj/Gene/Summary?g=MGP_CBAJ_G0022109&db=core) | 7.048065264 | 1.695293732 |
| ENSMUSG00000060586 | [H2-Eb1](http://asia.ensembl.org/mus_musculus_nzohlltj/Gene/Summary?g=MGP_NZOHlLtJ_G0024018&db=core) | 18.79370685 | 2.06371134 |
| ENSMUSG00000060969 | [Irx1](http://asia.ensembl.org/mus_musculus_nzohlltj/Gene/Summary?g=MGP_NZOHlLtJ_G0021168&db=core) | 23.7856977 | 54.9849927 |
| ENSMUSG00000061100 | [Retnla](http://asia.ensembl.org/mus_musculus_nzohlltj/Gene/Summary?g=MGP_NZOHlLtJ_G0023297&db=core) | 22.59843983 | 273.5810971 |
| ENSMUSG00000062248 | [Cks2](http://asia.ensembl.org/mus_musculus_nzohlltj/Gene/Summary?g=MGP_NZOHlLtJ_G0020978&db=core) | 47.5277028 | 11.29911781 |
| ENSMUSG00000062609 | [Kcnj15](http://asia.ensembl.org/mus_musculus_nzohlltj/Gene/Summary?g=MGP_NZOHlLtJ_G0023510&db=core) | 12.68555596 | 31.48402644 |
| ENSMUSG00000063011 | Msln | 91.96040864 | 186.1150468 |
| ENSMUSG00000063600 | [Egfem1](http://asia.ensembl.org/mus_musculus_lpj/Gene/Summary?g=MGP_LPJ_G0027056&db=core) | 54.47582632 | 19.56374303 |
| ENSMUSG00000063696 | Gm8730 | 30.89749366 | 0.353571878 |
| ENSMUSG00000064057 | [Scgb3a1](http://asia.ensembl.org/mus_musculus_nzohlltj/Gene/Summary?g=MGP_NZOHlLtJ_G0018668&db=core) | 4.473013047 | 143.1054885 |
| ENSMUSG00000066108 | [Muc5b](http://asia.ensembl.org/mus_musculus/Gene/Summary?g=ENSMUSG00000066108&db=core) | 0.101552502 | 2.656143416 |
| ENSMUSG00000066258 | Trim12a | 23.73977518 | 1.645410253 |
| ENSMUSG00000066362 | Rps13-ps1 | 0.100878361 | 73.67645539 |
| ENSMUSG00000066687 | [Zbtb16](http://asia.ensembl.org/mus_musculus/Gene/Summary?g=ENSMUSG00000066687&db=core) | 1.372776475 | 11.89244458 |
| ENSMUSG00000067017 | Capza1-ps1 | 0.550701256 | 6.2696003 |
| ENSMUSG00000069917 | Hba-a2 | 2310.623098 | 913.9563136 |
| ENSMUSG00000073940 | Hbb-bt | 1089.774442 | 253.6506401 |
| ENSMUSG00000076617 | Ighm | 22.19887593 | 3.189455692 |
| ENSMUSG00000079553 | Kifc1 | 34.85828074 | 5.757831584 |
| ENSMUSG00000091957 | Rps2-ps10 | 4.120476227 | 140.2221473 |
| ENSMUSG00000092274 | Neat1 | 83.66691197 | 192.6718092 |
| ENSMUSG00000096403 | Rnps1-ps | 0.067756003 | 10.26994827 |
| ENSMUSG00000096979 |  | 36.85427897 | 9.475465723 |
| ENSMUSG00000097451 |  | 5.289107501 | 27.48493287 |

**Table S2. Quantitative RT-PCR and genotyping primer sequences.**

| **Quantitative RT-PCR primers** | | | |
| --- | --- | --- | --- |
| **Gene** | **Forward (5'-3')** | | **Reverse (5'-3')** |
| *Aurkb* | CAGAAGGAGAACGCCTACCC | | GAGAGCAAGCGCAGATGTC |
| *Aurka* | CTGGATGCTGCAAACGGATAG | | CGAAGGGAACAGTGGTCTTAACA |
| *Cenpe* | CTTCAGTGGCTGTCTGTGTTC | | CCATCGCTCTGATAAATAGCGTT |
| *Cenpf* | GCTCAGCTTTTGCACCAGG | | AGGCGTAGTTCTAACTCAGTCAT |
| *Foxm1* | CTGATTCTCAAAAGACGGAGGC | | TTGATAATCTTGATTCCGGCTGG |
| *Cenpa* | CTCCAGTGTAGGCTCTCAGAC | | CTGAAAGGCTTCTTCCTGAACA |
| *Ccna2* | CTTGGCTGCACCAACAGTAA | | ATGACTCAGGCCAGCTCTGT |
| *Ccnd1* | GCATGTTCGTGGCCTCTAAGA | | GGTCTGTGCATGCTTGCG |
| *T1α* | TGCTACTGGAGGGCTTAATGA | | TGCTGAGGTGGACAGTTCCT |
| *Sftpc* | GAAGATGGCTCCAGAGAGCAT | | GGACTCGGAACCAGTATCATGC |
| *Scgb1a1* | CATGCTGTCCATCTGCTGC | | CTCTTGTGGGAGGGTATCC |
| *α-SMA* | TGACGCTGAAGTATCCGATAGA | | CGAAGCTCGTTATAGAAAGAGTGG |
| *PECAM* | CAAGCAAAGCAGTGAAGCTG | | TCTAACTTCGGCTTGGGAAA |
| *GAPDH* | GCACAGTCAAGGCCGAGAAT | | GCCTTCTCCATGGTGGTGAA |
| *Actin* | GGCTGTATTCCCCTCCATCG | | CCAGTTGGTAACAATGCCATGT |
| **Quantitative RT-PCR primers for ChIP assays** | | | |
| **Target site** | | **Forward (5'-3')** | **Reverse (5'-3')** |
| 1 | | ACAGCCCAGATTCTCTAGCA | TGAGGCCTGAGGTTCAGTCT |
| 2 | | CACCACACTCTGCTCACTCT | AAGCAGCTAGGTATGTGGCA |
| 3 | | TCAGAAAGGCGAAGACCCTG | AAGGGAGAGCTGCTTTGACC |
| 4 | | TTACACCGCGTTCTCAGCTC | CGCAGCCTCCTGTGATAACT |
| 5 | | TGTGCTCCCTCGCCATCAGACG | GCCCCAGCCCCCGAGGTGT |
| 6 | | TGGCTCACAGATCCAGGGTA | TGAGATGGGATGTGGCCCTA |
| **Genotyping primers** | | | |
| **Allele** | **Forward (5'-3')** | | **Reverse (5'-3')** |
| *Flox* | GTTTGTCGGAGGGCTCTGTTC | | GTCTGCTATCCCAGAATTCAAGAGG |
| *PRMT7KO* | GGGGTACCGCAGATGAGGAAGGAAAGTTCAGAC | | TCCCCGCGGCTGAACCCTCTCTCCAGCTCTAC |
| *TBX4* | AAGTCATTCCGCTGTGCTCT | | CAAAATCGTCAAGAGCGTCA |
| *Cre* | GACTCTGGTCAGAGATACCTGG | | CGAAATGTTCAGCACTACGCATAC |
| *SPC* | GACACATATAAGACCCTGGTCA | | AAAATCTTGCCAGCTTTCCCC |

**Table S3. Primary and secondary antibodies used in the study.**

| **Primary antibody** | **Source** | **Catalog #** | **Lot #** | **Dilution** |
| --- | --- | --- | --- | --- |
| Goat anti-Sox2 | Santa Cruz Biotechnology | sc-17320 | K2013 | 1:400 |
| Rabbit anti-Ki67 | Abcam | ab16667 | GR28243-1 | 1:400 |
| Mouse anti-Phospho-Histone H3(Ser10) (pH3) | Abcam | ab14955 | GR187373-1 | 1:400 |
| Rabbit anti-Sftpc | Santa Cruz Biotechnology | sc-13979 | E0912 | 1:400 |
| Goat anti-Sftpc | Santa Cruz Biotechnology | sc-7706 | H0114 | 1:400 |
| Rat anti-RAGE | R&D | MAB1179 | JYK0213101 | 1:400 |
| Rabbit anti-CC10 | Santa Cruz Biotechnology | sc-25555 | G0710 | 1:400 |
| Mouse anti-Acetylated-tubulin | Abcam | ab-24610 | GR281267-10 | 1:400 |
| Rabbit anti-Aqp5 | Abcam | ab104751 | GR34237-6 | 1:400 |
| Mouse anti-Abca3 | Abcam | ab24751 | GR133811-2 | 1:200 |
| Rabbit anti-CGRP | Sigma | C8189 | 012M4778 | 1:400 |
| Mouse anti-α-SMA(for sectional staining and western blotting) | Santa Cruz Biotechnology | sc-32251 | B0615 | 1:400 |
| Rabbit anti-PDGFRα(for sectional staining and western blotting) | Cell Signaling Technology | 3174 | 3 | 1:400 |
| Rabbit anti-Foxm1(for sectional staining and western blotting) | Santa Cruz Biotechnology | sc-502 | H1214 | 1:200 |
| Rabbit anti-H4R3me1 | Novus Biologicals | NB21-2011 | GR227766-1 | 1:1000 |
| Rabbit anti-H4R3me2s | Abcam | ab5823-200 | 97454 | 1:1000 |
| Rabbit anti-H3R2me2s | Novus Biologicals | NB21-1202 | A-1 | 1:1000 |
| Rabbit anti-H3R8me1 | Active Motif | 39673 | 31709001 | 1:1000 |
| Rabbit anti-H3R17me2 | UP-state | 07-214 | 25045 | 1:1000 |
| Rabbit anti-H3R8me2s | Novus Biologicals | NB21-1063 | A-1 | 1:1000 |
| Rabbit anti-H4 | Millipore | 05-858R | 09-271 | 1:5000 |
| Rabbit anti-H3 | UP-state | 07-690 | 30374 | 1:5000 |
| Rabbit anti-PRMT7 | GeneTex | GTX116570 | - | 1:1000 |
| Rabbit anti-GAPDH | GeneTex | GTX100118 | - | 1:5000 |
| **Secondary antibody** | **Source** | **Catalog #** | **Lot #** | **Dilution** |
| Donkey anti-goat 488 | Life Technologies | A11055 | 1687906 | 1:400 |
| Donkey anti-mouse 488 | Life Technologies | A21202 | 1644644 | 1:400 |
| Goat anti-rabbit 488 | Life Technologies | A11034 | 1705912 | 1:400 |
| Donkey anti-rabbit 555 | Life Technologies | A31572 | 1917920 | 1:400 |
| Donkey anti-rat 594 | Life Technologies | A21209 | 1661238 | 1:400 |
| HRP conjugate goat anti-rabbit | Thermo Fisher Scientific | 31460 | QC214563 | 1:5000 |
| HRP conjugate goat anti-mouse | Thermo Fisher Scientific | 31430 | QD216575 | 1:5000 |
